# Predicting Brain Amyloid Using Multivariate Morphometry Statistics, Sparse Coding, and Correntropy: Validation in 1,101 Individuals From the ADNI and OASIS Databases

**DOI:** 10.3389/fnins.2021.669595

**Published:** 2021-08-06

**Authors:** Jianfeng Wu, Qunxi Dong, Jie Gui, Jie Zhang, Yi Su, Kewei Chen, Paul M. Thompson, Richard J. Caselli, Eric M. Reiman, Jieping Ye, Yalin Wang

**Affiliations:** ^1^School of Computing, Informatics, and Decision Systems Engineering, Arizona State University, Tempe, AZ, United States; ^2^Institute of Engineering Medicine, Beijing Institute of Technology, Beijing, China; ^3^School of Cyber Science and Engineering, Southeast University, Nanjing, China; ^4^Banner Alzheimer’s Institute, Phoenix, AZ, United States; ^5^Imaging Genetics Center, Stevens Neuroimaging and Informatics Institute, University of Southern California, Marina del Rey, CA, United States; ^6^Department of Neurology, Mayo Clinic Arizona, Scottsdale, AZ, United States; ^7^Department of Computational Medicine and Bioinformatics, University of Michigan, Ann Arbor, MI, United States

**Keywords:** Alzheimer’s disease, hippocampal multivariate morphometry statistics, Dictionary and Correntropy-induced Sparse Coding, beta-amyloid burden, ADNI and OASIS database

## Abstract

Biomarker assisted preclinical/early detection and intervention in Alzheimer’s disease (AD) may be the key to therapeutic breakthroughs. One of the presymptomatic hallmarks of AD is the accumulation of beta-amyloid (Aβ) plaques in the human brain. However, current methods to detect Aβ pathology are either invasive (lumbar puncture) or quite costly and not widely available (amyloid PET). Our prior studies show that magnetic resonance imaging (MRI)-based hippocampal multivariate morphometry statistics (MMS) are an effective neurodegenerative biomarker for preclinical AD. Here we attempt to use MRI-MMS to make inferences regarding brain Aβ burden at the individual subject level. As MMS data has a larger dimension than the sample size, we propose a sparse coding algorithm, Patch Analysis-based Surface Correntropy-induced Sparse-coding and Max-Pooling (PASCS-MP), to generate a low-dimensional representation of hippocampal morphometry for each individual subject. Then we apply these individual representations and a binary random forest classifier to predict brain Aβ positivity for each person. We test our method in two independent cohorts, 841 subjects from the Alzheimer’s Disease Neuroimaging Initiative (ADNI) and 260 subjects from the Open Access Series of Imaging Studies (OASIS). Experimental results suggest that our proposed PASCS-MP method and MMS can discriminate Aβ positivity in people with mild cognitive impairment (MCI) [Accuracy (ACC) = 0.89 (ADNI)] and in cognitively unimpaired (CU) individuals [ACC = 0.79 (ADNI) and ACC = 0.81 (OASIS)]. These results compare favorably relative to measures derived from traditional algorithms, including hippocampal volume and surface area, shape measures based on spherical harmonics (SPHARM) and our prior Patch Analysis-based Surface Sparse-coding and Max-Pooling (PASS-MP) methods.

## Introduction

Alzheimer’s disease (AD) is a major public health concern with the number of affected individuals expected to triple, reaching 13.8 million by the year 2050 in the United States alone (Brookmeyer et al., 2007). Current therapeutic failures in patients with dementia due to AD may be due to interventions that are too late, or targets that are secondary effects and less relevant to disease initiation and early progression (Hyman, 2011). Preclinical AD is now viewed as a gradual process that begins many years before the onset of clinical symptoms. Measuring brain biomarkers and intervening at preclinical AD stages are believed to improve the probability of therapeutic success (Brookmeyer et al., 2007; Sperling et al., 2011a; Jack et al., 2016). In the A/T/N system – a recently proposed research framework for understanding the biology of AD – the presence of abnormal levels of beta-amyloid (Aβ) in the brain or cerebrospinal fluid (CSF) is used to define the presence of biological AD (Jack et al., 2016). An imbalance between production and clearance of Aβ occurs early in AD and is typically followed by the accumulation of tau protein tangles (another key pathological hallmark of AD) and neurodegeneration detectable on brain magnetic resonance imaging (MRI) scans (Hardy and Selkoe, 2002; Sperling et al., 2011a; Jack et al., 2016). Brain Aβ pathology can be measured using positron emission tomography (PET) with Aβ-sensitive radiotracers, or in CSF. Meanwhile, ongoing research has been focused on the harmonization over different tracers (Klunk et al., 2015), and the standardization of data acquisition and preprocessing (Su et al., 2015, 2018) for amyloid PET before they can be eventually implemented for clinical use. Even so, these invasive and expensive measurements are less attractive to subjects in preclinical stage and PET scanning is also not as widely available as MRI.

Blood-based biomarkers (BBBs) are somewhat effective for inferring Aβ burden in the brain and CSF, and are less expensive than imaging (Bateman et al., 2019; Janelidze et al., 2020; Palmqvist et al., 2020). Even so, structural MRI biomarkers are largely accessible, cost-effective, and widely used in AD imaging research as well as for clinical diagnosis. Consequently, there is great research interest in using MRI biomarkers to predict brain Aβ burden (Sperling et al., 2011a; Tosun et al., 2014, 2016; Pekkala et al., 2020). Tosun et al. (2014) combine MRI-based measures of cortical shape and cerebral blood flow to predict Aβ status for early-MCI individuals and achieve an 83% accuracy with the LASSO approach (least absolute shrinkage and selection operator). Pekkala et al. (2020) use brain MRI measures (volumes of the cortical gray matter, hippocampus, accumbens, thalamus, and putamen) to infer Aβ positivity in cognitively unimpaired (CU) subjects; they achieve a 0.70 area under the receiver operator curve (AUC) with their Disease State Index (DSI) algorithm. Although brain structural volumes are perhaps the most commonly used neuroimaging measures in AD research (Crivello et al., 2010; Reiter et al., 2017; Cacciaglia et al., 2018), surface-based subregional structure measures can offer advantages over volume measures as they contain more detailed and patient-specific shape information (Styner et al., 2004; Thompson et al., 2004; Morra et al., 2009; Qiu et al., 2009; Shen et al., 2009; Apostolova et al., 2010; Costafreda et al., 2011; Younes et al., 2014; Dong et al., 2019, 2020b; Ching et al., 2020).

Our prior studies (Wang et al., 2010, 2011; Shi et al., 2014) propose novel multivariate morphometry statistics (MMS) and apply them to analyze APOE4 dose effects on brain structures of non-demented and CU groups from the ADNI cohort (Shi et al., 2014; Li et al., 2016; Dong et al., 2019). Our proposed MMS approach uses multivariate tensor-based morphometry (mTBM) to encode morphometry along the surface tangent direction and radial distance (RD) to encode morphometry along the surface normal direction. This approach performs better for detecting clinically relevant group differences, relative to other TBM-based methods including those using the Jacobian determinant, the largest and smallest eigenvalues of the surface metric and the pair of eigenvalues of the Jacobian matrix (Wang et al., 2010, 2011). Our recent studies (Dong et al., 2019, 2020b) show that MMS outperforms volume measures for detecting hippocampal and ventricular deformations in groups at high risk for AD at the preclinical stage. Our other related work (Wu et al., 2018) has studied hippocampal morphometry in cohorts consisting of Aβ positive AD patients (Aβ+ AD) and Aβ negative CU subjects (Aβ− CU) using the MMS measure. We find significant Aβ+ AD vs. Aβ− CU group differences, using Hotelling’s *T*^2^ tests. As MMS have a high dimension, it is not suitable for classification research directly. Therefore, we apply a Patch Analysis-based Surface Sparse-coding and Max-Pooling (PASS-MP) system for a low-dimensional representation of hippocampal MMS, and the binary group random forest classification of Aβ+ AD and Aβ− CU, achieving an accuracy rate of 90.48%. These studies show that MMS can distinguish clinical groups with different Aβ status. We have also successfully applied PASS-MP for MMS-based AD cognitive scores and autism spectrum disorder predictions (Dong et al., 2020a; Fu et al., 2021).

In this work, we optimize the objective function of the PASS-MP system by introducing correntropy measure (Gui et al., 2017) and propose an improved sparse coding, dubbed as the Patch Analysis-based Surface Correntropy-induced Sparse-coding and Max-Pooling (PASCS-MP) method. PASCS-MP does not only take the advantage of the computational efficiency of PASS-MP in its new optimization strategy, but also effectively reduces the negative influence of non-Gaussian noise in the data, which tremendously improves the prediction accuracy. PASCS-MP is an unsupervised learning method to generate a low-dimensional representation for each sample. We leverage the novel PASCS-MP method on MMS to further explore hippocampal morphometry differences for the following contrasts at the individual subject level: (1) Aβ positive individuals with mild cognitive impairment (Aβ+ MCI) vs. Aβ negative individuals with mild cognitive impairment (Aβ− MCI) from ADNI, and (2) Aβ positive CU subjects (Aβ+ CU from ADNI and OASIS) vs. Aβ negative CU subjects (Aβ− CU from ADNI and OASIS). We apply the proposed PASCS-MP and a binary random forest classifier to classify individuals with different Aβ status. We hypothesize that our MMS-based PASCS-MP may provide stronger statistical power relative to traditional hippocampal volume, surface area and spherical harmonics (SPHARM) based hippocampal shape measurements, in predicting subjects’ Aβ status. We expect that the knowledge gained from this type of research will enrich our understanding of the relationship between hippocampal atrophy and AD pathology, and thus help in assessing disease burden, progression, and treatment effects.

## Subjects and Methods

### Subjects

Data used in the preparation of this article were obtained from the Alzheimer’s Disease Neuroimaging Initiative (ADNI) database^1^ and the Open Access Series of Imaging Studies (OASIS) database (Marcus et al., 2010). The ADNI was launched in 2003 as a public-private partnership, led by Principal Investigator Michael W. Weiner, MD. The primary goal of ADNI has been to test whether serial MRI, PET, other biological markers, and clinical and neuropsychological assessment can be combined to measure the progression of mild cognitive impairment (MCI) and early AD. For up-to-date information, see www.adni-info.org.

Table 1 shows demographic information we analyze from the ADNI and OASIS cohorts. From the ADNI cohort, we analyze 841 age and sex-matched subjects with florbetapir PET data and T1-weighted MR images, including 151 AD patients, 342 MCI, and 348 asymptomatic CU individuals. Among them, all the 151 AD patients, 171 people with MCI, and 116 CU individuals were Aβ positive. The remaining 171 MCI and 232 CU individuals were Aβ negative. From OASIS database, we analyze age-and-sex-matched 260 subjects with florbetapir PET data and T1-weighted MR images, including 52 Aβ positive CU and 208 Aβ negative CU. To match the age and sex to the control group, we randomly select subjects from the majority group millions of times. For each selected group and control group, we analyze the age with *t*-test and sex with Chi-square test. We first select the groups, of which the *p*-value of Chi-squared test is 1. Among these groups, we select the one with the largest *p*-value of age. In this way, we try to minimize the statistical difference in age and sex. The *p*-values of Chi-squared test for sex and the *p*-values of *t*-test for age are shown in the columns after sex and age in Table 1.

**TABLE 1:** Demographic information for the subjects we study from the ADNI and OASIS cohorts.

Database	Group	Sex (M/F)	*p*-Value	Age	*p*-Value	MMSE	Centiloid
ADNI (*n* = 841)	*A*β + *AD* (*n* = 151)	79/72		74.6 ± 7.8		22.6 ± 3.1	86.3 ± 27.4
	*A*β + *MCI* (*n* = 171)	92/79	1.00	74.1 ± 7.4	0.90	27.7 ± 1.7	76.8 ± 26.4
	*A*β − *MCI* (*n* = 171)	92/79		74.0 ± 7.4		28.3 ± 1.6	8.9 ± 14.9
	*A*β + *CU* (*n* = 116)	45/71	1.00	75.9 ± 6.1	0.78	28.9 ± 1.1	71.1 ± 26.4
	*A*β − *CU* (*n* = 232)	90/142		75.7 ± 6.3		29.0 ± 1.3	7.5 ± 14.5
OASIS (*n* = 260)	*A*β + *CU* (*n* = 52)	22/30	1.00	70.5 ± 7.5	0.08	29.0 ± 1.3	71.4 ± 20.9
	*A*β − *CU* (*n* = 208)	88/120		68.5 ± 6.8		29.0 ± 1.3	8.5 ± 9.5

*Values are mean ± SD where applicable.*

In addition to each MRI scan, we also analyze centiloid measures (Navitsky et al., 2018) from florbetapir PET data in ADNI and OASIS. Operationally, the *positivity* of Aβ biomarkers is defined using standard cut-offs, with some efforts to reconcile differences among different Aβ radiotracers using a norming approach called the centiloid scale (Klunk et al., 2015; Rowe et al., 2017). ADNI florbetapir PET data are processed using AVID pipeline (Navitsky et al., 2018), and OASIS florbetapir PET data are processed using PUP (Lee et al., 2013; Su et al., 2015). Both are converted to the centiloid scales according to their respective conversion equations (Navitsky et al., 2018; Su et al., 2019). A centiloid cutoff of 37.1 is used to determine Aβ positivity, this threshold corresponds to pathologically determined moderate to frequent plaques (Fleisher et al., 2011).

### Proposed Pipeline

**FIGURE 1:**
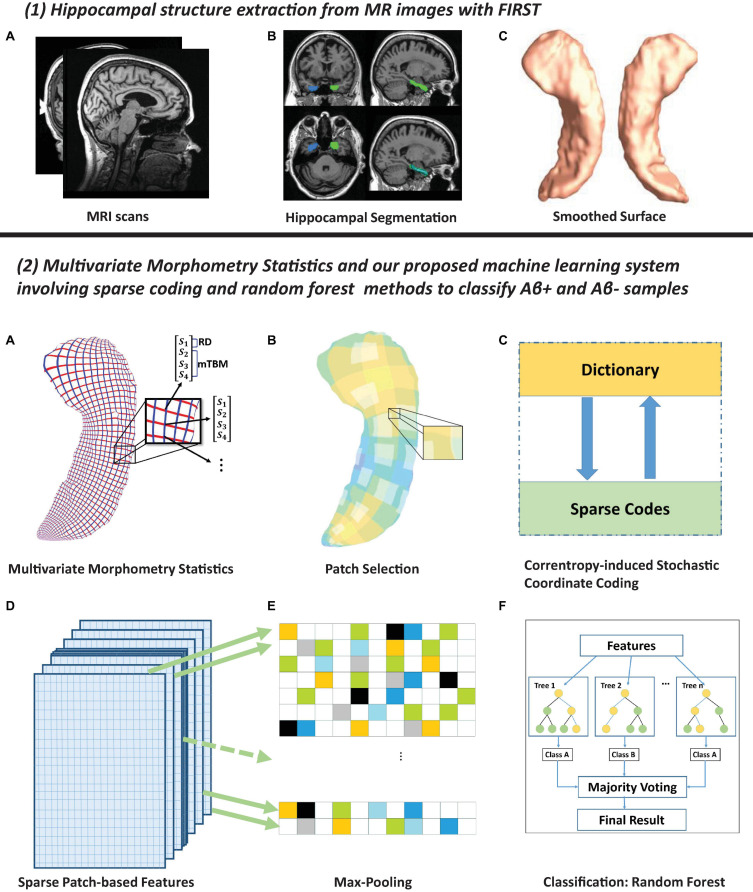
Panel **(1)** shows hippocampal surfaces generated from brain MRI scans. In subfigure **(A)** of panel **(2)**, surface-based multivariate morphometry statistics (MMS) are calculated after fluid registration of surface coordinates across subjects. MMS is a 4 × 1 vector on each vertex, including radial distance (scalar) and multivariate tensor-based morphometry (3 × 1 vector). In subfigures **(B,C)**, we randomly select patches on each hippocampal surface and generate a sparse code for each patch with our novel Patch Analysis-based Surface Correntropy-induced Sparse-coding (PASCS) method. In subfigures **(D,E)**, we apply the max pooling operation to the learned sparse codes to generate a new representation (a vector) for each subject. In subfigure **(F)**, we train binary random forest classifiers on these representations and validate them with 10-fold cross-validation.

This work develops the PASCS-MP framework to predict individual Aβ burden (see Figure 1 for the processing pipeline). In panel (1), hippocampal structures are segmented from registered brain MR images with FMRIB’s Integrated Registration and Segmentation Tool (FIRST) from the FMRIB Software Library (FSL) (Patenaude et al., 2011; Paquette et al., 2017). Hippocampal surface meshes are constructed with the marching cubes algorithm (Lorensen and Cline, 1987). In panel (2), hippocampal surfaces are parameterized with the holomorphic flow segmentation method (Wang et al., 2007). After the surface fluid registration algorithm, the hippocampal MMS features are calculated at each surface point. We propose a PASCS-MP and classification system to refine and classify MMS patches in individuals with different Aβ status. We randomly select patches on each hippocampal surface and generate a sparse code for each patch with our novel PASCS. Next, we adopt a Max-pooling operation on the learned sparse codes of these patches to generate a new representation (a vector) for each subject. Finally, we train binary random forest classifiers on individual sparse codes in people with different Aβ status; we validate them with 10-fold cross-validation. The whole system is publicly available.^2^

#### Image Processing

Firstly, we use FIRST (Patenaude et al., 2011) to segment the original MRI data and map the hippocampus substructure. After obtaining a binary segmentation of the hippocampus, we use a topology-preserving level set method (Han et al., 2003) to build surface models. Based on that, the marching cubes algorithm (Lorensen and Cline, 1987) is applied to construct triangular surface meshes. Then, to reduce the noise from MR image scanning and to overcome partial volume effects, surface smoothing is applied consistently to all surfaces. Our surface smoothing process consists of mesh simplification using *progressive meshes* (Hoppe, 1996) and mesh refinement by the Loop subdivision surface method (Loop, 1987). Similar procedures adopted in a number of our prior studies (Wang et al., 2010, 2012; Colom et al., 2013; Luders et al., 2013; Monje et al., 2013; Shi et al., 2013a, b, 2015) have shown that the smoothed meshes are accurate approximations to the original surfaces, with a higher signal-to-noise ratio (SNR).

To facilitate hippocampal shape analysis, we generate a conformal grid (150×100) on each surface, which is used as a canonical space for surface registration. On each hippocampal surface, we compute its conformal grid with a holomorphic 1-form basis (Wang et al., 2007, 2010). We adopt surface conformal representation (Shi et al., 2013a, 2015) to obtain surface geometric features for automatic surface registration. This consists of the conformal factor and mean curvature, encoding both intrinsic surface structure and information on its three-dimensional embedding. After we compute these two local features at each surface point, we compute their summation and then linearly scale the dynamic range of the summation into the range 0–255, to obtain a feature image for the surface. We further register each hippocampal surface to a common template surface. With surface conformal parameterization and conformal representation, we generalize the well-studied image fluid registration algorithm (Bro-Nielsen and Gramkow, 1996; D’Agostino et al., 2003) to general surfaces. Furthermore, most of the image registration algorithms in the literature are not symmetric, i.e., the correspondences between the two images depending on which image is assigned as the deforming image and which is the non-deforming target image. An asymmetric algorithm can be problematic as it tends to penalize the expansion of image regions more than shrinkage (Rey et al., 2002). Thus, in our system, we further extend the surface fluid registration method to an inverse-consistent framework (Leow et al., 2005). The obtained surface registration is diffeomorphic. For details of our inverse-consistent surface fluid registration method, we refer to (Shi et al., 2013a).

#### Surface-Based Morphometry Feature Extraction

After parameterization and registration, we establish a one-to-one correspondence map between hippocampal surfaces. This makes it effective for us to compare and analyze surface data. Besides, each surface has the same number of vertices (150×100) as shown in panel (2) of Figure 1. The intersection of the red curve and the blue curve is a surface vertex, and at each vertex, we adopt two features, the RD and the surface metric tensor used in mTBM. The RD (a scalar at each vertex) represents the thickness of the shape at each vertex to the medical axis (Pizer et al., 1999; Thompson et al., 2004), this reflects the surface differences along the surface normal directions. The medial axis is determined by the geometric center of the isoparametric curve on the computed conformal grid (Wang et al., 2011). The axis is perpendicular to the isoparametric curve, so the thickness can be easily calculated as the Euclidean distance between the core and the vertex on the curve. The mTBM statistics (a 3×1 vector at each vertex) have been frequently studied in our prior work (Shi et al., 2013b, 2015; Wang et al., 2009, 2010). They measure local surface deformation along the surface tangent plane and show improved signal detection sensitivity relative to more standard tensor-based morphometry (TBM) measures computed as the determinant of the Jacobian matrix (Wang et al., 2013). RD and mTBM jointly form a new feature, known as the surface MMS. Therefore, MMS is a 4×1 vector at each vertex. The surface of the hippocampus in each brain hemisphere has 15, 000 vertices, so the feature dimensionality for each hippocampus in each subject is 60,000.

#### Surface Feature Dimensionality Reduction

The above mentioned vertex-wise surface morphometry feature, MMS, is a high-fidelity measure to describe the local deformation of the surface and can provide detailed localization and visualization of regional atrophy or expansion (Yao et al., 2018) and development (Thompson et al., 2000). However, the high dimensionality of such features is likely to cause problems for classification. Feature reduction methods proposed by Davatzikos et al. (2008) and Sun et al. (2009) may ignore the intrinsic properties of a structure’s regional morphometry. Therefore, we introduce the following feature reduction method for the vertex-wise surface morphometry features.

The surface MMS feature dimension is typically much larger than the number of subjects, i.e., the so-called *high dimension-small sample problem*. To extract useful surface features and reduce the dimension before making predictions, this work first randomly generates square windows on each surface to obtain a collection of small image patches with different amounts of overlap. In our prior AD studies (Zhang et al., 2016a, b; Wu et al., 2018), we discuss the most suitable patch size and number. Therefore, in this work, we adopt the same optimal experimental settings, as 1, 008 patches (patch size = 10×10 vertices) for each subject (*504* patches for each side of the hippocampal surface). As these patches are allowed to overlap, a vertex may be contained in several patches. The zoomed-in window in subfigure B of panel (2) in Figure 1 [panel (2), B] shows overlapping areas on selected patches. After that, we use the technique of sparse coding and dictionary learning (Mairal et al., 2009) to learn meaningful features. Dictionary learning has been successful in many image processing tasks as it can concisely model natural image patches. In this work, we propose a novel sparse coding and dictionary learning method with an *l*_1_-regularized correntropy loss function named *Correntropy-induced Sparse-coding (CS)*, which is expected to improve the computational efficiency compared to Stochastic Coordinate Coding (SCC) (Lin et al., 2014). Formally speaking, correntropy is a generalized similarity measure between two scalar random variables U and V, which is defined by 𝒱_σ_(*U*,*V*) = *𝔼*𝒦_σ_(*U*,*V*). Here, 𝒦_σ_ is a Gaussian kernel given by 𝒦_σ_(*U*,*V*) = exp{−(u − v)^2^/σ^2^} with the scale parameter σ > 0, (u-v) being a realization of (U, V) (Feng et al., 2015; Gui et al., 2017). Utilizing the correntropy measure as a loss function will reduce the negative influence of non-Gaussian noise in the data.

Classical dictionary learning techniques (Olshausen and Field, 1997; Lee et al., 2007) consider a finite training set of feature maps, *X* = (*x*_1_,*x*_2_,*x*_*n*_) in *R*^*p*×*n*^. In our study, *X* is the set of MMS features from *n* surface patches of all the samples. All the MMS features on each surface patch, *x*_*i*_, is reshaped to a *p*-dimensional vector. And we desire to generate a new set of sparse codes, *Z* = (*z*_1_,*z*_2_,*z*_*n*_) in *R*^*m*×*n*^ for these features. Therefore, we aim to optimize the empirical cost function as Eq. 1.

(1) $$f\,\left(D,z_{i}\right)≜\sum_{i=1}^{n}l\,\left(x_{i},D,z_{i}\right)$$

where *D* ∈ *R*^*p*×*m*^ is the dictionary and *z*_*i*_ ∈ *R^m^* is the sparse code of each feature vector. *l*(*x*_*i*_,*D*,*z*_*i*_) is the loss function that measures how well the dictionary *D* and the sparse code *z*_*i*_ can represent the feature vector *x*_*i*_. Then, *x*_*i*_ can be approximated by *x*_*i*_ = *Dz*_*i*_. In this way, we convert the *p*-dimensional feature vector, *x*_*i*_, to a *m*-dimensional sparse code, *z*_*i*_, where *m* is the dimensionality of the sparse code and the dimensionality could be arbitrary. In this work, we introduce the correntropy measure (Gui et al., 2017) to the loss function and define the *l*_*1*_-sparse coding optimization problem as Eq. 2

(2) $$\underset{D,z_{i}}{\text{min}}\frac{1}{2}\,\sum_{i=1}^{n}e\,x\,p\,\left(-\frac{{∥D\,z_{i}-x_{i}∥}_{2}^{2}}{\sigma^{2}}\right)+\lambda \,\sum_{i=1}^{n}{∥z_{i}∥}_{1}$$

where λ is the regularization parameter, σ is the kernel size that controls all properties of correntropy. ∥⋅∥_2_ and ∥⋅∥_1_are the *l*_*2*_-norm and *l*_*1*_-norm and *exp()* represents the exponential function. The first part of the loss function measures the degree of the image patches’ goodness and the correntropy may help remove outliers. Meanwhile, the second part is well known as the *l*_*1*_ penalty (Fu, 1998) that can yield a sparse solution for *z*_*i*_ and select robust and informative features. Specifically, there are *m* columns (atoms) in the dictionary *D* and each atom is *d*_*j*_ ∈ *R^p^*,*j* = 1, 2,…,*m*. To avoid *D* from being arbitrarily large and leading to arbitrary scaling of the sparse codes, we constrain each *l*_*2*_-norm of each atom in the dictionary no larger than one. We will let *C* become the convex set of matrices verifying the constraint as Eq. 3.

(3) $$\text{C}≜\left\{D\in R^{p\times m}s.t.\forall j=1,2,,m,d_{j}^{T}d_{j}\leq 1\right\}$$

Note that, the empirical problem cost *f*(*D*,*z*_*i*_) is not convex when we jointly consider the dictionary *D* and the coefficients *Z*. But the function is convex concerning each of the two variables, *D*, and *Z*, when the other one is fixed. Since it takes much time to solve *D* and *Z* when dealing with large-scale data sets and a large-size dictionary, we adopt the framework in the stochastic coordinate coding (SCC) algorithm (Lin et al., 2014), which can dramatically reduce the computational cost of the sparse coding, while keeping a comparable performance.

To solve this optimization problem, we reformulate the first part of the equation by the half-quadratic technique (Nikolova and Ng, 2006) and then the objective can be solved as the minimization problem Eq. 4:

(4) $$\underset{D,z_{i}}{\text{min}\,\frac{1}{2}}\sum_{i=1}^{n}h_{i}\,{∥D\,z_{i}-x_{i}∥}_{2}^{2}+\lambda \,\sum_{i=1}^{n}{∥z_{i}∥}_{1},$$

$$h_{i}=\exp\left(-\frac{{∥D\,z_{i}-x_{i}∥}_{2}^{2}}{\sigma^{2}}\right).$$

Here the auxiliary variable, *h*_*i*_, will be updated in each update iteration. At each iteration, we update *D* and *Z* alternately, which means we firstly fix *D* and update the sparse code *Z* with coordinated descent (CD) and then fix Z to update the dictionary *D via* stochastic gradient descent (SGD).

As our optimization method is stochastic, we only update the sparse code and dictionary with only one signal for each iteration. In the following paragraphs, we will discuss the optimization in one iteration with only one signal. If a signal, *x* = (*x*_1_,*x*_2_,*x*_*p*_)*^T^* ∈ *R^p^*, is given, we first update its corresponding sparse code, *z* = (*z*_1_,*z*_2_,…,*z*_*m*_), *via* CD. Let *z*_*l*_ denote the *l*-th entry of *z* and *d*_*kl*_ represents the *k*-th item of *d*_*l*_. *d*_*l*_ is the *l*-th atom/column of the dictionary *D*. Then, we can calculate the partial derivative of *z*_*l*_ in the first part of the function, *f*(*D*,*z*_*i*_), as Eq. 5

(5) $$\frac{\partial }{\partial z_{l}}\,c\,\left(D,\text{z}\right)=\frac{\partial }{\partial z_{l}}\,\frac{1}{2}\,h\,{∥D\,z-x∥}_{2}^{2}=-h\,\sum_{k=1}^{p}d_{k\,l}\,\left(x_{k}-\sum_{r=1}^{m}d_{k\,r}\,z_{r}\right)$$

$$=-h\,\sum_{k=1}^{p}d_{k\,l}\,\left(x_{k}-\sum_{r\neq l}^{m}d_{k\,r}\,z_{r}-d_{k\,l}\,z_{l}\right)$$

$$=-h\,\sum_{k=1}^{p}d_{k\,l}\,\left(x_{k}-\sum_{r\neq l}^{m}d_{k\,r}\,z_{r}\right)+h\,z_{l}\,\sum_{k=1}^{p}{\left(d_{k\,l}\right)}^{2}$$

$$=-\rho_{l}+h\,z_{l}\,\upsilon_{l}$$

where $\rho_{l}=h\,\sum_{k=1}^{p}d_{k\,l}\,\left(x_{k}-\sum_{r\neq l}^{m}d_{k\,r}\,z_{r}\right)$, $\upsilon_{l}=\sum_{k=1}^{p}{\left(d_{k\,l}\right)}^{2}$ and *h* is the auxiliary variable for the signal. Since we normalize the atom, *d*_*l*_, in each iteration, υ_*l*_ can be ignored. Then, we compute the subdifferential of the lasso loss function and equate it to zero to find the optimal solution as follows:

(6) $$\frac{\partial }{\partial z_{l}}\,f\,\left(D,\text{z}\right)=\frac{\partial }{\partial z_{l}}\,c\,\left(D,\text{z}\right)+\frac{\partial }{\partial z_{l}}\,\lambda \,{∥z∥}_{1}$$

$$=-\rho_{l}+h\,z_{l}\,\upsilon_{l}+\frac{\partial }{\partial z_{l}}\,\lambda \,{∥z∥}_{1}=0$$

Then, according to the derivative of the *l*_1_-norm, we can have the following equations.

(7) $$\left\{ \begin{array}{cc} -\rho \,l+h\,z_{l}\,\upsilon_{l}-\lambda =0 & \text{if}\,Z_{l}<0\\ -\rho \,l-\lambda \leq 0\leq -\rho_{l}+\lambda & \text{if}\,Z_{l}=0\\ -\rho \,l+h\,z_{l}\,\upsilon_{l}+\lambda =0 & \text{if}\,Z_{l}>0 \end{array}\right.$$

Finally, we can get the soft thresholding function as:

(8) $$z_{l}=\left\{ \begin{array}{cc} \frac{\rho_{l}+\lambda }{h\,\upsilon_{l}} & \text{for}\,\rho_{l}<-\lambda \\ 0 & \text{for}-\lambda \leq \rho_{l}\leq \lambda \\ \frac{\rho_{l}-\lambda }{h\,\upsilon_{l}} & \text{for}\,\rho_{l}>\lambda \end{array}\right.$$

**FIGURE 2:**
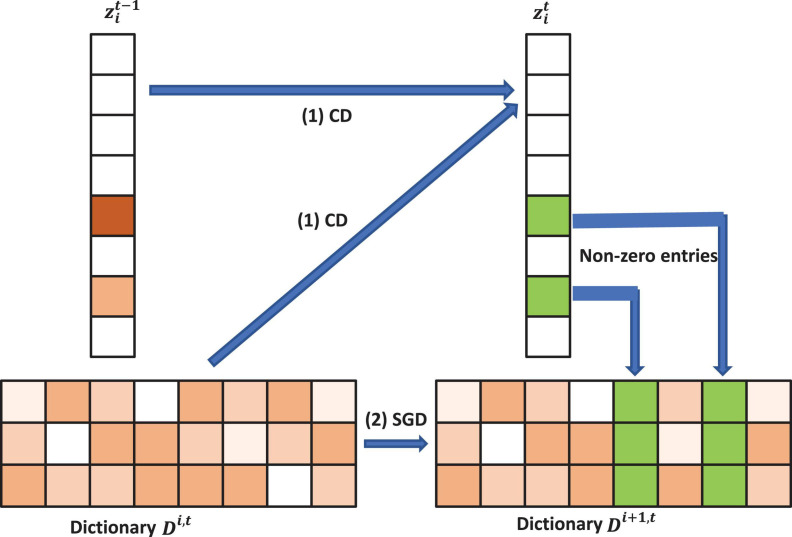
Illustration of one iteration of the proposed Patch Analysis-based Surface Correntropy-induced Sparse-coding (PASCS) algorithm. The input is many 10 × 10 patches on each surface based on our multivariate morphometry statistics (MMS). With an image patch *x*_*i*_, PASCS performs one step of coordinate descent (CD) to find the support and the sparse code. Meanwhile, PASCS performs a few steps of CD on supports (non-zero entries) to obtain a new sparse code $z_{i}^{k}$. Then, PASCS updates the supports (*green boxes in the figure*) of the dictionary by stochastic gradient descent (SGD) to obtain a new dictionary *D*^*i*1,*t*^. Here, *t* represents the *t-*th epoch; *i* represents the *i*-th patch.

After we update the sparse code, we propose the following strategy to accelerate the convergence for updating the dictionary *D*. The atom, *d*_*l*_ will stay unchanged if *z*_*l*_ is zero since ∇*_dl_* = *h*(*Dz*−*x*)*z*_*l*_ = 0. Otherwise, as shown in Figure 2, we can update the *l*-th atom of the dictionary *D* as *d*_*l*_←*d*_*l*_−γ_*l*_*h*(*Dz*−*x*)*z*_*l*_. γ_*l*_ is the learning rate provided by an approximation of the Hessian: *R*←*R* + *zz^T^* and γ_*l*_ is given by 1/*r*_*ll*_, where *r*_*ll*_ is the item at the *l*-th row and *l*-th column of the Hessian matrix *R*. The pseudo-code of the model was shown in Algorithm 1, dubbed as PASCS.

**Algorithm 1:** Patch analysis-based surface correntropy-induced sparse-coding.

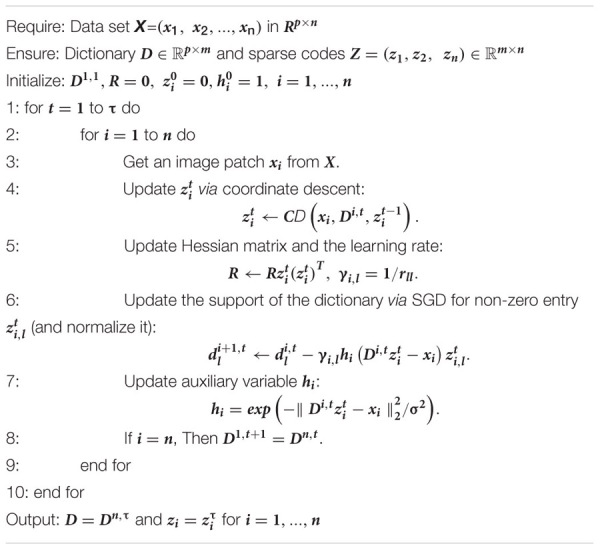

#### Pooling and Classification

After we get the sparse code (the dimension is *m*) for each patch, the dimensionality of sparse codes for each subject is still too large for classification, which is m×1,008. Therefore, we apply Max-pooling to reduce the feature dimensionality for each subject. Max-pooling (Boureau et al., 2010) is a way of taking the most responsive node of a given region of interest and serves as an important layer in the convolutional neural network architecture. In this work, we compute the maximum value of a particular feature over all sparse codes of a subject and generate a new representation for each subject, which is an *m*-dimensional vector. These summary representations are much lower in dimension, compared to using all the extracted surface patch features; this can improve results generalizability *via* less over-fitting.

With these dimension-reduced features, we choose the random forest algorithm (Liaw and Wiener, 2002) for the binary classification. Random forests are a combination of tree predictors such that each tree depends on the values of a random vector sampled independently and with the same distribution for all trees in the forest. This algorithm adopts a learning process called *feature bagging*. In this process, we select a random subset of the features several times and then train a decision tree for each subset. If some features are strong predictors of the response, they will be selected in many decision trees and this makes them correlated. In comparison with decision trees, random forests have the same bias but lower variance, which means they can overcome the drawback of overfitting caused by a small data set. For our sparse surface features, when the size of the training set becomes small, diversification becomes more subtle, and the method can better detect these subtle differences. In this project, we use the random forest classifier in the *scikit-learn* package^3^ with the default settings. Besides, under the imbalanced-data condition (such as 116 Aβ+ CU and 232 Aβ− CU in the ADNI data set), the classifier tends to classify all the training data into the major class, as it aims to maximize training accuracy. Therefore, we adopt *random undersampling* (Dubey et al., 2014) to balance the numbers of training subjects in the two classes. All the experiments in this work use the same setups for the random forest classifier and random undersampling.

### Performance Evaluation Protocol

Before using hippocampal MMS features for Aβ status classification, we need to apply PASCS-MP to extract sparse codes from these high dimensional MMS features. The performance of PASCS-MP has a close relationship to four key parameters: the patch size, the dimensionality of the learned sparse coding, the regularization parameter for the *l*_*1*_-norm (λ), and the kernel size (σ) in the exponential function (see Eq. 2). Patch-based analysis has been widely used for image segmentation and classification (Kao et al., 2020). Leveraging patches in our MMS can preserve well the properties of the regional morphometry of the hippocampal surface since the vertices that carry strong classification power are always clustered on the surface and a set of such vertices typically has a stronger classification ability compared to using just a single vertex. However, the size of the set of such vertices is unknown. Therefore, we select the vertices by randomly selecting the same number of square patches with different sizes and compared the performance of the final classification accuracy for the different patch sizes. The dimensionality of the learned sparse coding (*m*) is also the dimensionality of the representation for each subject. The model might miss some significant information if the dimensionality is too low. Also, the representations will contain too much redundant information when the dimensionality is too large. The regularization parameter for the *l*_1_-norm (λ) will control the sparsity of the learned sparse codes. A suitable regularization parameter will select significant features meanwhile reducing noise. The kernel size in the exponential function controls all properties of correntropy. Correntropy is directly related to the probability of how similar two random variables are in a neighborhood of the joint space controlled by the kernel bandwidth, i.e., the kernel bandwidth acts as a zoom lens, controlling the *observation window* over which similarity is assessed. This adjustable window provides an effective mechanism to eliminate the detrimental effect of outliers (Liu et al., 2007).

Thus, we adopt 10-fold cross-validation to evaluate the classification accuracy on another dataset from ADNI 2 with a series of key parameter candidates and select the optimal parameter setups. The detailed information about the dataset and the key parameter candidates will be introduced in next section. For the 10-fold cross-validation, we randomly shuffle and split the dataset into ten groups. We take one group as the test data set and use the remaining groups to train a model. Then, the candidate model is evaluated using the test data. In this way, we can get a predicted class label for all the samples. Then, the output of each classification experiment is compared to the ground truth, and the accuracy is computed to indicate how many class labels are correctly identified. The key parameters with the highest classification accuracies are selected.

Once we get an optimized PASCS-MP model, we can compare the performances of MMS, volume, and surface area measurements for classifying individuals of different Aβ status. We use the volume from the left and right hippocampi (i.e., hippocampi in each brain hemisphere) as two features to train the classifier instead of adding them together. The same classification strategy is applied to surface areas from both sides. Moreover, we will compare the classification performances based on PASCS-MP, PASS-MP (Zhang et al., 2016b, 2017b), and SPHARM (Chung et al., 2007, 2008; Shi et al., 2013a). We evaluate these classification performances with the same 10-fold cross-validation method. Four performance measures: the Accuracy (ACC), Balanced Accuracy (B-ACC), Specificity (SPE), and Sensitivity (SEN) are computed (Hinrichs et al., 2011; Ritter et al., 2015; Zhang et al., 2017b; Bhagwat et al., 2018; Salvatore et al., 2018). We also compute the area-under-the-curve (AUC) of the receiver operating characteristic (ROC) (Fan et al., 2008; La Joie et al., 2013; Bhagwat et al., 2018; Nakamura et al., 2018). By considering these performance measures, we expect the proposed system integrating MMS, PASCS-MP and the binary random forest classifier to perform better than similar classification strategies for identifying individuals with different Aβ status.

## Results

### Key Parameter Estimations for the PASCS-MP Method

To apply PASCS-MP method on hippocampal MMS, four parameters need to be empirically assigned, namely: the patch size, the dimensionality of the learned sparse coding, the regularization parameter for the *l*_*1*_-norm (λ) and the kernel size (σ) in the exponential function. Selecting suitable parameters will lead to superior performance in refining lower dimensional MMS representations related to AD pathology. With 10-fold cross-validation, these key parameters are evaluated from PASCS-MP based classification performance on 109 AD patients and 180 CU subjects of ADNI-2 cohort. To avoid data leakage, these subjects are not used in the following study of Aβ burden classification.

**FIGURE 3:**
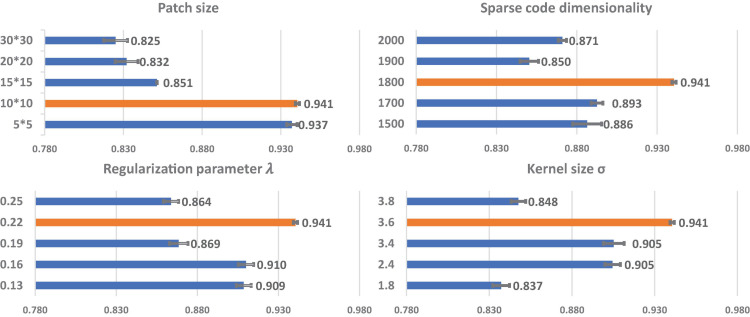
The relationship of each parameter to classification accuracy. The *y*-axis represents the value for each parameter. The orange bars represent the classification performances using the optimal parameters. Each bar represents the average and 95% confidence interval of classification accuracy.

We perform grid search on the data set to explore the optimal parameter settings. In Figure 3, we only illustrate part of the classification accuracy for different values of each parameter in grid search since the combinations of four different parameters will lead to 5^4^ results. For each parameter setting, we also repeat 10-fold cross-validation five times, and the average and 95% confidence interval of the accuracy are shown in Figure 3. When we evaluate one parameter, we fix the rest parameters. For example, in the first bar chart in Figure 3, we try different patch sizes including 5 × 5, 10 × 10, 15 × 15, 20 × 20. and 30 × 30 while we fix the sparse code dimensionality as to 1,800, and set λ to 0.22, and σ to 3.6. By testing varied sets of parameters, we find that the optimal patch size is 10 × 10, the optimal sparse code dimensionality is 1,800, the optimal λ is 0.22 and the optimal σ is 3.6 and these optimal parameters will be adopted in the study of Aβ burden classification.

### Classification of Aβ Burden

To explore whether there is a significant gain in classification power with our new system, based on our surface MMS, we generate two different kinds of sparse codes with our previous framework (PASS-MP) (Zhang et al., 2016b, 2017a; Fu et al., 2021) and the new framework (PASCS-MP). The parameter settings for the two sparse coding methods are the same. Additionally, we apply the popular SPHARM method (Chung et al., 2008; Shi et al., 2013a) to calculate hippocampal shape features. Based on these three kinds of feature sets, we apply the random forest classifier to detect individuals with different Aβ status. Moreover, we also examine the classification performances using hippocampal MMS, surface area and volume measures. These classification performances are evaluated using ACC, B-ACC, SPE, and SEN. For each binary classification of ADNI cohort, we repeat the 10-fold cross-validation five times; the mean and 95% confident interval of the evaluation measures are calculated as (Vanwinckelen and Blockeel, 2012) and shown in the middle three columns of Table 2.

**TABLE 2:** Classification results for four contrasts.

	Aβ+ AD vs. Aβ− CU	Aβ+ MCI vs. Aβ− MCI	Aβ+ CU vs. Aβ− CU (ADNI)	Aβ+ CU vs. Aβ− CU (OASIS)
**Area**				
ACC	0.68 ± 0.01	0.55 ± 0.02	0.54 ± 0.01	0.47
B-ACC	0.69 ± 0.02	0.55 ± 0.02	0.54 ± 0.02	0.43
SPE	0.66 ± 0.02	0.54 ± 0.02	0.55 ± 0.02	0.49
SEN	0.71 ± 0.03	0.56 ± 0.03	0.53 ± 0.04	0.37
**Volume**				
ACC	0.71 ± 0.01	0.53 ± 0.02	0.50 ± 0.03	0.51
B-ACC	0.72 ± 0.01	0.53 ± 0.01	0.50 ± 0.03	0.52
SPE	0.68 ± 0.01	0.52 ± 0.01	0.51 ± 0.02	0.54
SEN	0.75 ± 0.01	0.54 ± 0.02	0.49 ± 0.04	0.50
**SPHARM**				
ACC	0.71 ± 0.02	0.56 ± 0.02	0.52 ± 0.02	0.60
B-ACC	0.71 ± 0.02	0.56 ± 0.03	0.51 ± 0.04	0.60
SPE	0.74 ± 0.02	0.61 ± 0.03	0.56 ± 0.03	0.61
SEN	0.68 ± 0.04	0.51 ± 0.03	0.46 ± 0.05	0.60
**PASS-MP**				
ACC	0.79 ± 0.01	0.73 ± 0.02	0.71 ± 0.02	0.74
B-ACC	0.79 ± 0.01	0.73 ± 0.02	0.70 ± 0.03	0.73
SPE	0.78 ± 0.02	0.75 ± 0.02	0.73 ± 0.03	0.74
SEN	0.79 ± 0.01	0.72 ± 0.03	0.67 ± 0.03	0.73
**PASCS-MP**				
ACC	0.91 ± 0.01	0.89 ± 0.01	0.79 ± 0.02	0.81
B-ACC	0.91 ± 0.01	0.89 ± 0.01	0.79 ± 0.03	0.80
SPE	0.91 ± 0.01	0.91 ± 0.01	0.80 ± 0.02	0.82
SEN	0.90 ± 0.01	0.88 ± 0.01	0.79 ± 0.05	0.79

*Values are mean ± 95% confident interval where applicable.*

**FIGURE 4:**
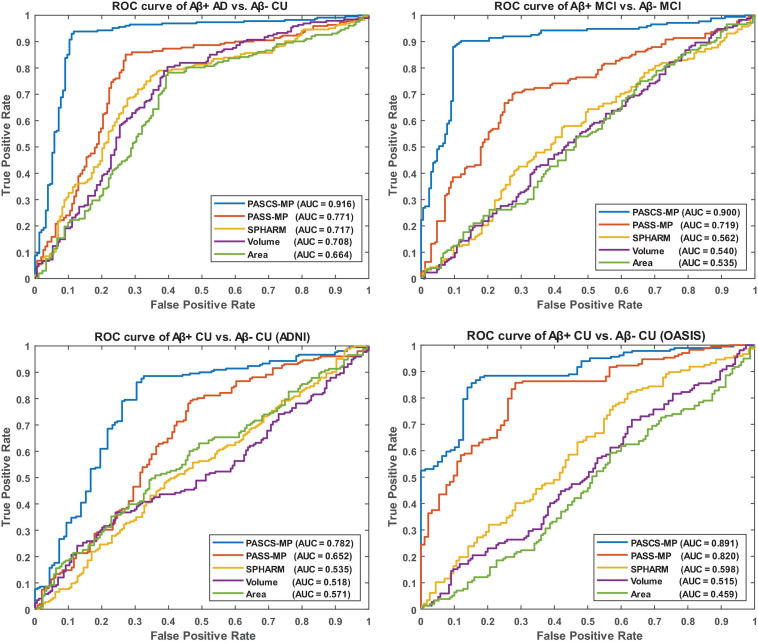
Receiver operating characteristic curves for the classification tasks, Aβ+ AD vs. Aβ– CU, Aβ+ MCI vs. Aβ– MCI, Aβ+ CU vs. Aβ– CU (ADNI), and Aβ+ CU vs. Aβ– CU (OASIS). OASIS is used as an external validation set for the model trained by ADNI CU.

We leverage the OASIS dataset as an external validation set to further evaluate the performance of our new framework. We firstly generate new representations with our proposed PASCS-MP for all the CU subjects from ADNI and OASIS cohorts. Then, we train a binary random forest model on the ADNI dataset and test it with the OASIS dataset. Since there is no cross-validation here, there is no confident interval in the last column of Table 2. We also compute the AUC of the ROC. The ROC curve and AUC for these classification tasks are illustrated in Figure 4. This comparison analysis classification performance shows that the combination of PASCS-MP and hippocampal MMS measures have superior performance when detecting individuals with different Aβ status, compared to other similar methods.

## Discussion

In this article, we propose a novel surface feature dimension reduction scheme, PASCS-MP, to correlate the hippocampus MMS with different levels of Aβ burden in individual subjects. We develop a hippocampal structure-based Aβ burden prediction system that involves hippocampal MMS computing, sparse coding and classification modules. We apply the proposed system on two independent datasets, ADNI and OASIS. We have two main findings. Firstly, the hippocampal surface-based MMS measure practically encodes a great deal of neighboring intrinsic geometry information that would otherwise be inaccessible or overlooked in classical hippocampal volume and surface area measures. Experimental results show that the MMS measure provides better classification accuracy than hippocampal volume, surface area measures and SPHARM for detecting the relationships between hippocampal deformations and Aβ positivity. Secondly, we propose a novel sparse coding method, PASCS-MP. It has all the advantages of our previous proposed PASS-MP (Zhang et al., 2016a, b) and improves the follow-up classification performance compared to PASS-MP.

### Comparison Analysis of MRI, PET, and BBB

Amyloid plaques, together with neurofibrillary tangles, are among the earliest signs of AD, appearing before any cognitive impairment and brain structure changes. Measuring Aβ burden at preclinical AD stages is believed to facilitate identifying individuals appropriate for a given intervention and improving the probability of therapeutic trial success (Brookmeyer et al., 2007; Jack et al., 2016; Sperling et al., 2011a). Brain Aβ pathology can be measured using PET with amyloid directly in the brain, CSF measures, or, more recently, BBBs, which are showing great promise.

Nevertheless, assessment of Aβ pathology using CSF or PET scans can easily become inefficient due to the degree of their acceptance, invasiveness, costs, and/or PET facility availability (Tosun et al., 2016, 2014; Ansart et al., 2020; Pekkala et al., 2020). Developing computational models for detecting Aβ pathology based on less invasive, less costly, and more readily available procedures could help identify a target population with a high prevalence of Aβ pathology. With or without more affordable BBBs as an alternative for inferring Aβ burden in the brain (Bateman et al., 2019; Janelidze et al., 2020; Palmqvist et al., 2020), especially after their diagnosis viabilities are further reduced, sMRI scans will always be an option that is largely accessible, cost-effective, and widely used as a standard-of-care procedure. Structural MRI will also identify pathologies that AD BBB’s do not assess, e.g., stroke, tumor, and subdural hematoma. Therefore, a patient will get an MRI scan whether or not they also get BBB. Furthermore, our proposed framework will not only work on AD, but also other diseases like autism spectrum disorder (Fu et al., 2021), major depressive disorder (Yao et al., 2020). Overall, our work will provide important research tools and good insights for computational neuropathology research.

### Comparison Analysis of Hippocampal MMS, Volume, and Surface Area

The hippocampus is a primary target region for studying early AD progression. Its structure can be measured using the widely used overall hippocampal volume, surface area, and our proposed hippocampal MMS. Our prior studies (Shi et al., 2011; Wang et al., 2011; Li et al., 2016; Dong et al., 2019) show that hippocampal MMS performs robustly in distinguishing clinical groups at different AD risk levels. In particular, we previously found that hippocampal MMS can detect *APOE4* gene dose effects on the hippocampus during the preclinical stage, while the hippocampal volume measure cannot (Dong et al., 2019). A study by Wu et al. (2018) demonstrates that hippocampal MMS performs better than traditional hippocampal volume measures in classifying 151 Aβ+ AD and 271 Aβ− CU subjects.

This work evaluates the performance of the above three hippocampal measurements for predicting Aβ status at the individual subject level. Classification results (see Table 2 and Figure 4) show that hippocampal MMS has better performance as measured by ACC, SPE, SEN, and AUC. These results validate our hypothesis that hippocampal MMS-based analysis methods provide improved statistical accuracy than hippocampal volume and surface area measures in predicting the subjects with different Aβ status in the AD continuum. Our prior work (Wang et al., 2011) shows that MMS may offer a surrogate biomarker for PET/CSF Aβ biomarkers. This work further shows it can be used to classify brain Aβ burden on an individual basis.

### Comparative Analysis of PASCS-MP, PASS-MP, and SPHARM

The MMS measure for brain structures performs well in clinical group comparisons (Wang et al., 2013; Shi et al., 2014, 2015; Li et al., 2016; Yao et al., 2018; Dong et al., 2019, 2020b), and as we have shown, it has the potential to further be applied for individual Aβ classification. To achieve this goal, we need to solve the challenge that the MMS dimension is usually much larger than the number of subjects, i.e., the so-called *high dimension, small sample size problem*. A reasonable solution is to reduce the feature dimension. Existing feature dimension reduction approaches include feature selection (Jain and Zongker, 1997; Fan et al., 2005), feature extraction (Mika et al., 1999; Jolliffe, 2002; Guyon et al., 2008) and sparse learning methods (Donoho, 2006; Vounou et al., 2010; Wang et al., 2013). In most cases, information is lost when mapping data into a lower-dimensional space. By defining a better lower-dimensional subspace, this information loss can be limited. Sparse coding (Lee et al., 2007; Mairal et al., 2009) has been previously proposed to learn an over-complete set of basis vectors (also called a *dictionary*) to represent input vectors efficiently and concisely (Donoho and Elad, 2003). Sparse coding has been shown to be effective for many tasks such as image imprinting (Moody et al., 2012), image deblurring (Yin et al., 2008), super-resolution (Yang et al., 2008), classification (Mairal et al., 2009), functional brain connectivity (Lv et al., 2015, 2017), and structural morphometry analysis (Zhang et al., 2017a).

Our previous studies (Zhang et al., 2016a, b, 2017a) propose a PASS-MP method, consisting of sparse coding (Lee et al., 2007; Mairal et al., 2009), and Max-pooling (LeCun et al., 2015), for surface feature dimension reduction. PASS-MP has excellent impressive performance for the sparse coding of our MMS features. Our prior studies successfully apply these sparse codes in detecting individual brain structure abnormalities and obtain state-of-art performance (Wu et al., 2018; Dong et al., 2020a; Fu et al., 2021).

Even so, there typically exists non-Gaussian and localized sources of noise in surface-based morphometry features, this can dramatically influence the learned dictionary and further lead to poor sparse coding based on the loss function of PASS-MP. The correntropy measure is a very robust method for correcting such sources of noise (Nikolova and Ng, 2006; Liu et al., 2007; He et al., 2012). In this article, we improve upon the PASS-MP method by introducing correntropy measures into the loss function (Gui et al., 2017). Therefore, our proposed sparse coding method, PASCS-MP, incorporates all the advantages of PASS-MP and meanwhile improves the classification performance. We also test SPHARM-based hippocampal shape features as they have frequently been studied in prior AD research (e.g., Gerardin et al., 2009; Cuingnet et al., 2011; Gutman et al., 2013). In such an approach, we use a series of SPHARM to model the shapes of the hippocampus segmented by FSL. The SPHARM coefficients are computed using SPHARM-PDM (Spherical Harmonics-Point Distribution Model) software developed by the University of North Carolina and the National Alliance for Medical Imaging Computing (Styner et al., 2006). The classification features are based on these SPHARM coefficients, which are represented by two sets of three-dimensional SPHARM coefficients for each subject (in fact, one set for the hippocampus in each brain hemisphere). In Gerardin et al. (2009), they use a feature selection step because the subject groups are much smaller (fewer than 30 subjects in each group). When the number of subjects is small, the classifier can be more sensitive to uninformative features. In the current study, the number of subjects is relatively large, so a feature selection step is less necessary and may increase the risk of overfitting. We adopt the same approach in Cuingnet et al. (2011), who chose to avoid this selection step. The classification results (see Table 2 and Figure 4) based on PASCS-MP, PASS-MP, and SPHARM meet our expectation that the classification performances based on PASCS-MP have an apparent improvement measured by ACC, B-ACC, SPE, SEN, and AUC.

### Aβ Burden Prediction Using MRI Biomarkers

Beta-amyloid accumulation is a major feature of AD neuropathology (Brier et al., 2016; Cummings, 2019). Detecting it early and accurately provides a potential opportunity for effective therapeutic interventions before the advanced stages of AD (Tosun et al., 2014). Compared to PET and CSF Aβ measurement techniques, MRI is less expensive (than PET) and less invasive (than both PET and lumbar puncture). AD-related biomarker studies (Sperling et al., 2011b; Jack and Holtzman, 2013; Jack et al., 2018) have shown that abnormal brain Aβ accumulation typically precedes detectable structural brain abnormalities. There is emerging literature using MRI biomarkers to predict brain Aβ burden, and hippocampal structural measurement is one of the major predictors (Tosun et al., 2014, 2016; Ansart et al., 2020; Pekkala et al., 2020). Tosun et al. (2014) applied LASSO penalized logistic regression classifier to MRI-based voxel-wise anatomical shape variation measures and cerebral blood flow measures to predict Aβ positivity in 67 people with early MCI (34 Aβ+); the classification accuracy was 83%. Ansart et al. (2020) applied LASSO feature selection and a random forest classifier to MRI-based cortical thickness and hippocampal volume measures to classify 596 people with MCI scanned as part of ADNI MCI (375 Aβ+); the AUC was 0.80. Trzepacz et al. (2016) also leveraged logistic regression models with elastic net and hippocampal volume to predict Aβ status in 252 EMCI patients (120 Aβ+) and 136 LMCI patients (92 Aβ+), the AUCs were 0.70 and 0.71. Our proposed classification framework has a higher ACC = 89% or AUC = 0.90 than each of these two studies (Tosun et al., 2014; Ansart et al., 2020) for predicting Aβ status in people with MCI. Of the studies predicting Aβ positivity in CUs, Ansart et al. (2020) applied LASSO feature selection and random forest classifier to MRI-derived cortical thickness and hippocampal volume measures to classify 431 ADNI CUs (162 Aβ+) and 318 INSIGHT CUs (88 Aβ+); the AUCs were 0.59 and 0.62, respectively. Pekkala et al. (2020) used the DSI machine learning algorithm and MRI-based biomarkers (total cortical and gray matter volumes, hippocampus, accumbens, thalamus, and putamen volumes) to predict Aβ burden in 48 CUs (20 Aβ+); the AUC was 0.78. Tosun et al. (2021) utilized MRI-score extracted from MRI by a trained deep learning model to classify the groups with different Aβ status in 269 CU patients (100 Aβ+). The AUC and ACC were 0.74 and 0.67. Our proposed classification framework has AUC = 0.78 on 348 ADNI CUs (116 Aβ+) and AUC = 0.89 on 260 OASIS CUs (52 Aβ+). Table 3 and Figure 4 present the AUC or ACC values from this work and from similar studies predicting Aβ positivity using brain MRI biomarkers. Compared to these similar studies, our proposed classification system only uses hippocampal structural features but still consistently outperforms other recently published methods for predicting Aβ positivity in people with MCI and CUs.

**TABLE 3:** Studies to impute Aβ status from MRI biomarkers in key clinical groups in AD research.

Method	Subjects (Aβ+/−)	MRI biomarkers	ACC	AUC
PASCS-MP-Random forest classifier (this work)	342 ADNI MCI (171/171)	Hippocampal multivariate morphometry statistics (MMS)	0.89 ± 0.01	0.90
	348 ADNI CU (116/232)		0.79 ± 0.02	0.78
	260 OASIS CU (52/208)		0.81	0.89
LASSO penalized logistic regression classifier (Tosun et al., 2014)	67 early MCI (34/33)	Voxel-wise anatomical shape variation measures and cerebral blood flow (including frontoparietal cortical, hippocampal regions, among others)	0.83 ± 0.03	
LASSO feature selection and random forest classifier (Ansart et al., 2020)	596 ADNI MCI (375/221)	Cortical thickness and hippocampal volume		0.80
	431 ADNI CU (162/269)			0.59
	318 INSIGHT CU (88/230)			0.62
Disease State Index machine learning algorithm (Pekkala et al., 2020)	48 CU (20/28)	Total cortical and gray matter volumes, hippocampus, accumbens, thalamus, and putamen volumes		0.78
Logistic regression analyses including elastic net classifier (Trzepacz et al., 2016)	ADNI EMCI (120/132)	Hippocampal volume		0.70
	ADNI LMCI (92/44)			0.71
Random forest (Tosun et al., 2021)	ADNI CU (109/224)	MRI-score extracted by a deep learning model	0.67 ± 0.04	0.74

### Parameter Selection and Accuracy

Sparse coding is a representation learning method to generate a sparse representation of the input data in the form of the linear combination of sparse code and a dictionary. Grid search is an effective way to select rational regularization parameters and sparse code dimensionality. However, the accuracy of classification with different parameter settings may not always follow a perfect Gaussian distribution. In Figure 3, the regularization parameter (0.19) and the sparse code dimensionality (1900) have an erratic effect on the classification accuracy. In some other similar work for sparse coding (Xu et al., 2014; Plenge et al., 2015; Quan et al., 2016), the regularization parameter and the sparse code dimensionality also had similar erratic effects on the classification accuracy. It may be due to data insufficiency. Even so, the two cohorts studied in this work arguably had one of the largest imaging data in exiting amyloid prediction research. Further research is warranted to carefully study the relationship between parameter settings and the accuracy results.

### Limitations and Future Work

Despite the promising results are obtained by applying our proposed Aβ positivity classification framework, there are two important caveats. First, when applying the PASCS-MP method to refine MMS, we generally cannot visualize the selected features. To some extent, this decreases the interpretability of the effects, although it is still possible to visualize statistically significant regions as in our prior group difference studies (Shi et al., 2013a; Wang et al., 2013). However, in our recent work (Wu et al., 2020), instead of randomly selecting patches to build the initial dictionary, we use group lasso screening to select the most significant features first. Therefore, the features used in sparse coding may be visualized on the surface map. In the future, we will incorporate this idea into the PASCS-MP framework to make it more interpretable. Second, this work only applies hippocampal MMS to predict Aβ positivity. In future work, we plan to introduce more AD risk factors (such as demographic information, genetic information, and clinical assessments) (Tosun et al., 2014; Ansart et al., 2020; Pekkala et al., 2020), and more AD regions of interest (ROIs; e.g., ventricles, entorhinal cortex, and temporal lobes) (Brier et al., 2016; Foley et al., 2017; Dong et al., 2020b) into our proposed framework; these additional features are expected to improve the Aβ positivity prediction.

## Conclusion

In this article, we explore the association between hippocampal structures and Aβ positivity on two independent databases using our hippocampal MMS, PASCS-MP method and a random forest classifier. Compared to traditional hippocampal shape measures, MMS have superior performance for predicting Aβ positivity in the AD continuum. Besides, the proposed PASCS-MP outperforms our previous sparse coding method (PASS-MP) on refining MMS features. Compared to similar studies, this work achieves state-of-the-art performance when predicting Aβ positivity based on MRI biomarkers. In the future, we plan to apply this proposed framework to other AD ROIs and further improve the comprehensibility of the framework by visualizing morphometry features selected in the classification.

## Data Availability Statement

The original contributions presented in the study are included in the article/Supplementary Material, further inquiries can be directed to the corresponding author/s.

## Ethics Statement

The studies involving human participants were reviewed and approved by the Alzheimer’s Disease Neuroimaging Initiative. The patients/participants provided their written informed consent to participate in this study.

## Author Contributions

JW: methodology, investigation, formal analysis, and writing–original draft. QD: conceptualization, investigation, formal analysis, and writing–original draft. JG, JZ, YS, PT, and JY: methodology. KC: methodology and review and editing. RC and ER: review and editing. YW: conceptualization, investigation, supervision, funding acquisition, and writing–review and editing. All authors contributed to the article and approved the submitted version.

## Conflict of Interest

The authors declare that the research was conducted in the absence of any commercial or financial relationships that could be construed as a potential conflict of interest.

## Publisher’s Note

All claims expressed in this article are solely those of the authors and do not necessarily represent those of their affiliated organizations, or those of the publisher, the editors and the reviewers. Any product that may be evaluated in this article, or claim that may be made by its manufacturer, is not guaranteed or endorsed by the publisher.

## Footnotes

1
adni.loni.usc.edu

2
http://gsl.lab.asu.edu/software/pass-mp/

3
https://scikit-learn.org/
