# Supplementary material for: Predicting Brain Amyloid Using Multivariate Morphometry Statistics, Sparse Coding, and Correntropy: Validation in 1,101 Individuals From the ADNI and OASIS Databases
Source: Front Neurosci. 2021 Aug 6;15:669595. doi: 10.3389/fnins.2021.669595 (PMC8377280; doi:10.3389/fnins.2021.669595)
Supplement: Supplementary file 1 [file Data_Sheet_1.PDF]

ADNI database (Subject id: **Sub_ID,** Visit code: **Vis_code)**

Sub_ID	Vis_Code	Sub_ID	Vis_Code	Sub_ID	Vis_Code	Sub_ID	Vis_Code
002_S_0295	v06	021_S_4659	v01	051_S_1072	m48	126_S_4458	v01
002_S_0413	v06	021_S_4718	v03	051_S_4980	v03	126_S_4494	v03
002_S_0729	m48	021_S_4857	v01	051_S_5005	v03	126_S_4507	v01
002_S_1261	m48	021_S_5129	v02	052_S_0671	m60	126_S_4675	v01
002_S_1268	m48	021_S_5177	v02	052_S_1346	m48	126_S_4686	v03
002_S_1280	m48	021_S_5236	v02	052_S_1352	m48	126_S_4891	v01
002_S_2043	sc	021_S_5237	v02	052_S_2249	sc	126_S_4896	v01
002_S_2073	sc	021_S_6312	sc	052_S_4626	v01	126_S_5214	v02
002_S_2073	v51	022_S_0096	m60	052_S_4807	v01	127_S_0259	v11
002_S_4171	v01	022_S_0130	m60	052_S_4885	v01	127_S_0260	m60
002_S_4213	v02	022_S_2087	sc	052_S_4944	v01	127_S_0925	m48
002_S_4219	v01	022_S_2167	sc	052_S_4945	v01	127_S_2213	sc
002_S_4225	v02	022_S_2379	sc	052_S_4959	v03	127_S_2234	sc
002_S_4237	v01	022_S_4173	v02	052_S_5062	v03	127_S_4198	v02
002_S_4251	v01	022_S_4196	v02	053_S_2357	sc	127_S_4210	v01
002_S_4262	v02	022_S_4266	v02	053_S_4557	v01	127_S_4240	v01
002_S_4270	v02	022_S_4291	v02	053_S_4578	v02	127_S_4301	v01
002_S_4447	v01	022_S_4320	v02	053_S_4661	v01	127_S_4500	v03
002_S_4521	v01	022_S_4444	v01	053_S_5070	v03	127_S_4624	v01
002_S_4654	v01	022_S_4444	v41	053_S_5202	v02	127_S_4624	v21
002_S_4746	v01	022_S_4805	v01	053_S_5272	v02	127_S_4645	v02
002_S_4799	v01	022_S_4922	v01	053_S_5287	v02	127_S_4843	v02
002_S_4799	v21	023_S_0031	m60	053_S_6598	sc	127_S_4844	v01
002_S_5018	v03	023_S_0061	m60	057_S_0934	m60	127_S_4940	v03
002_S_5230	v02	023_S_0331	v06	057_S_1269	m48	127_S_4992	v03
002_S_5256	v02	023_S_0887	m48	057_S_2398	sc	127_S_5028	v03
002_S_6007	scmri	023_S_1046	m48	057_S_4909	v01	127_S_5056	v03
002_S_6066	scmri	023_S_1190	v06	057_S_5199	v02	127_S_5058	v03
002_S_6103	scmri	023_S_2068	sc	057_S_5292	v02	127_S_5095	v03
002_S_6456	scmri	023_S_4034	v01	057_S_5295	v02	127_S_5132	v02
003_S_0981	m48	023_S_4035	v01	067_S_0056	m60	127_S_5185	v02
003_S_1057	m48	023_S_4122	v01	067_S_0059	m60	127_S_5200	v02
003_S_1074	m48	023_S_4164	v02	067_S_0257	v21	127_S_5218	v02
003_S_2374	sc	023_S_4241	v01	067_S_2195	sc	127_S_5228	v02
003_S_4081	v02	023_S_4243	v01	067_S_2196	sc	128_S_0227	m48
003_S_4119	v02	023_S_4501	v03	067_S_2301	sc	128_S_0229	m48
003_S_4152	v03	023_S_4502	v01	067_S_2301	init	128_S_0272	m48
003_S_4288	v02	023_S_4796	v01	067_S_2304	sc	128_S_0545	m48
003_S_4354	v01	023_S_5120	v03	067_S_4054	v01	128_S_0863	m48
003_S_4373	v03	023_S_6346	scmri	067_S_4072	v01	128_S_1043	v06
003_S_4524	v01	023_S_6356	sc	067_S_4184	v01	128_S_1408	m36
003_S_4555	v02	023_S_6369	sc	067_S_4212	v41	128_S_2002	sc
003_S_4892	v03	023_S_6535	sc	067_S_4728	v03	128_S_2003	sc
003_S_4900	v02	023_S_6547	scmri	067_S_4767	v21	128_S_2011	sc

003_S_5154	v02
003_S_5165	v03
003_S_5187	v03
003_S_5209	v02
003_S_6258	sc
003_S_6260	scmri
003_S_6268	sc
003_S_6432	sc
005_S_0553	m48
005_S_0610	m48
005_S_4168	v01
005_S_4185	v01
005_S_4910	v03
005_S_5119	v03
005_S_6084	scmri
005_S_6427	sc
006_S_0498	m48
006_S_0731	init
006_S_1130	m48
006_S_4153	v03
006_S_4192	v03
006_S_4357	v02
006_S_4449	v02
006_S_4515	v01
006_S_4546	v03
006_S_4679	v01
006_S_4713	v41
006_S_4867	v03
006_S_4960	v01
006_S_5153	v02
007_S_0101	v06
007_S_0698	m48
007_S_1206	v06
007_S_2106	sc
007_S_4272	v01
007_S_4387	v02
007_S_4467	v01
007_S_4568	v03
007_S_4611	v01
007_S_4620	v02
007_S_4637	v02
007_S_4911	v03
007_S_5196	v03
007_S_5265	v02
009_S_0751	v11
009_S_0842	m48
009_S_1030	m48
024_S_0985	m48
024_S_2239	sc
024_S_4084	v02
024_S_4158	v02
024_S_4674	v01
024_S_4905	v03
024_S_5054	v03
024_S_5290	v02
027_S_0074	m60
027_S_0118	m60
027_S_0120	m60
027_S_0835	m60
027_S_2183	sc
027_S_2219	sc
027_S_2336	sc
027_S_4729	v01
027_S_4757	v01
027_S_4801	v03
027_S_4802	v03
027_S_4869	v01
027_S_4873	v01
027_S_4919	v01
027_S_4936	v01
027_S_4955	v01
027_S_4962	v03
027_S_4964	v03
027_S_4966	v01
027_S_5083	v02
027_S_5093	v02
027_S_5109	v02
027_S_5118	v02
027_S_5127	v02
027_S_5170	v02
027_S_5197	v02
027_S_5277	v02
027_S_5288	v02
029_S_0845	m48
029_S_1218	m48
029_S_1318	m48
029_S_2376	sc
029_S_2395	sc
029_S_4279	v02
029_S_4290	v02
029_S_4307	v03
029_S_4327	v01
029_S_4385	v02
029_S_4652	v02
067_S_5159	v02
067_S_5205	v03
067_S_5212	v02
068_S_2168	sc
068_S_2171	sc
068_S_2184	sc
068_S_2184	init
068_S_2193	sc
068_S_2194	sc
068_S_2248	sc
068_S_2315	sc
068_S_4061	v01
068_S_4067	v01
068_S_4134	v01
068_S_4174	v02
068_S_4217	v01
068_S_4332	v01
068_S_4332	init
068_S_4424	v02
068_S_4431	v01
068_S_4859	v03
068_S_4968	v03
068_S_5146	v03
070_S_4692	v03
070_S_4708	v01
070_S_4719	v03
070_S_4793	v01
070_S_5040	v02
072_S_0315	v06
072_S_1380	v06
072_S_2026	sc
072_S_2027	sc
072_S_2037	sc
072_S_2083	sc
072_S_2164	sc
072_S_4226	v01
072_S_4390	v01
072_S_4391	v02
072_S_4445	v01
072_S_4465	v01
072_S_4522	v01
072_S_4610	v01
072_S_4613	v01
072_S_4769	v01
072_S_4871	v01
072_S_5207	v02
073_S_0089	m48
128_S_2057	sc
128_S_2123	sc
128_S_2130	sc
128_S_2220	sc
128_S_4553	v01
128_S_4607	v02
128_S_4609	v02
128_S_4636	v01
128_S_4653	v01
128_S_4671	v01
128_S_4742	v01
128_S_4745	v01
128_S_4774	v03
128_S_4842	v01
128_S_4842	v41
128_S_5066	v01
128_S_5123	v03
129_S_2332	init
129_S_4073	v01
129_S_4220	v01
129_S_4287	v01
129_S_4371	v02
129_S_4396	v02
129_S_4422	v02
129_S_6228	scmri
129_S_6288	scmri
129_S_6457	scmri
129_S_6459	scmri
130_S_0232	m60
130_S_0289	m60
130_S_0505	v06
130_S_0886	v06
130_S_0969	v21
130_S_2373	sc
130_S_2391	sc
130_S_2403	sc
130_S_4294	v01
130_S_4343	v02
130_S_4352	v02
130_S_4415	v01
130_S_4417	v01
130_S_4542	v01
130_S_4589	v03
130_S_4605	v01
130_S_4641	v03
130_S_4660	v03
130_S_4730	v03

009_S_2208	sc	029_S_5135	v01	073_S_0311	v06	130_S_4883	v01
009_S_4324	v01	029_S_5135	v21	073_S_0746	v06	130_S_4925	v01
009_S_4388	v02	029_S_5158	v02	073_S_2153	sc	130_S_4971	v03
009_S_4530	v01	029_S_6289	scmri	073_S_2182	sc	130_S_4982	v03
009_S_4543	v41	031_S_0618	m48	073_S_2190	sc	130_S_4984	v03
009_S_4814	v01	031_S_0830	m48	073_S_4155	v02	130_S_4990	v03
009_S_4903	v01	031_S_0867	m48	073_S_4216	v01	130_S_4997	v03
009_S_5000	v01	031_S_2018	sc	073_S_4259	v01	130_S_5006	v03
009_S_5027	v03	031_S_2022	sc	073_S_4300	v01	130_S_5059	v03
009_S_5037	v03	031_S_4005	v01	073_S_4311	v01	130_S_5142	v02
009_S_5176	v02	031_S_4024	v03	073_S_4312	v01	130_S_5175	v02
009_S_5252	v03	031_S_4029	v01	073_S_4382	v02	130_S_5231	v03
009_S_6212	scmri	031_S_4032	v02	073_S_4393	v02	130_S_5258	v02
010_S_4345	v02	031_S_4203	v01	073_S_4540	v01	131_S_0123	m60
011_S_0021	m60	031_S_4218	v02	073_S_4559	v02	135_S_4281	v01
011_S_0023	m60	031_S_4474	v02	073_S_4853	v03	135_S_4309	v01
011_S_2274	sc	031_S_4476	v01	073_S_4986	v01	135_S_4406	v01
011_S_4075	v02	031_S_4496	v02	073_S_5090	v03	135_S_4446	v02
011_S_4105	v02	031_S_4590	v01	073_S_5227	v02	135_S_4489	v01
011_S_4120	v02	031_S_4721	v01	082_S_2121	sc	135_S_4566	v02
011_S_4222	v02	032_S_0214	m60	082_S_2307	sc	135_S_4657	v03
011_S_4278	v02	032_S_0479	v06	082_S_4208	v02	135_S_4863	v03
011_S_4547	v01	032_S_0677	v51	082_S_4244	v01	135_S_4954	v03
011_S_4827	v03	032_S_2240	sc	082_S_4339	v02	135_S_5015	v03
011_S_4845	v03	032_S_4277	v02	082_S_4428	v02	135_S_5273	v02
011_S_4893	v01	032_S_4348	v02	082_S_5029	v03	135_S_5275	v03
011_S_4906	v03	032_S_4386	v02	082_S_5184	v03	136_S_0107	v06
011_S_4912	v03	032_S_4429	v02	082_S_5278	v02	136_S_0186	v06
011_S_4949	v03	032_S_4755	v03	082_S_5282	v02	136_S_0873	m48
011_S_6367	scmri	032_S_4921	v02	082_S_6197	scmri	136_S_4269	v02
011_S_6465	scmri	032_S_5263	v02	082_S_6287	scmri	136_S_4408	v01
012_S_4012	v01	032_S_5289	v02	082_S_6564	scmri	136_S_4433	v02
012_S_4026	v02	032_S_6211	scmri	094_S_2201	v11	137_S_0301	v06
012_S_4128	v01	032_S_6279	scmri	094_S_2367	sc	137_S_0668	v11
012_S_4545	v02	032_S_6293	scmri	094_S_4089	v03	137_S_0972	m48
012_S_4987	v01	032_S_6294	scmri	094_S_4162	v01	137_S_0994	m48
012_S_5121	v02	033_S_0734	v06	094_S_4234	v02	137_S_1414	v11
013_S_1186	m48	033_S_0741	v06	094_S_4282	v03	137_S_4211	v03
013_S_4268	v01	033_S_0906	m48	094_S_4503	v02	137_S_4258	v03
013_S_4395	v01	033_S_0920	m48	094_S_4560	v02	137_S_4299	v01
013_S_4579	v02	033_S_0923	m48	094_S_4630	v01	137_S_4331	v01
013_S_4580	v02	033_S_1016	m48	098_S_0160	v06	137_S_4351	v01
013_S_4595	v01	033_S_1098	m48	098_S_0171	v06	137_S_4351	v21
013_S_4616	v02	033_S_4176	v02	098_S_0172	v06	137_S_4466	v02
013_S_4917	v01	033_S_4179	v02	098_S_0667	m48	137_S_4482	v02
013_S_4985	v01	033_S_4505	v02	098_S_0896	m48	137_S_4536	v01
013_S_5137	v02	033_S_4508	v02	098_S_2047	sc	137_S_4587	v02

013_S_5171	v02	033_S_5013	v03	098_S_4003	v02	137_S_4596	v01
014_S_0658	m48	033_S_5017	v03	098_S_4018	v02	137_S_4623	v01
014_S_2308	sc	033_S_5087	v03	098_S_4050	v02	137_S_4632	v02
014_S_4039	v03	033_S_5198	v02	098_S_4201	v03	137_S_4672	v03
014_S_4058	v01	033_S_5259	v02	098_S_4215	v03	137_S_4678	v01
014_S_4079	v01	035_S_0156	init	099_S_0051	v06	137_S_4756	v03
014_S_4080	v02	035_S_0997	m48	099_S_0291	v06	137_S_4816	v01
014_S_4093	v02	035_S_2199	sc	099_S_0352	v06	137_S_4852	v01
014_S_4263	v01	035_S_4256	v01	099_S_2042	sc	141_S_0717	m48
014_S_4328	v01	035_S_4414	v01	099_S_2205	sc	141_S_0767	m48
014_S_4576	v02	035_S_4582	v01	099_S_4022	v01	141_S_1004	v06
014_S_4577	v02	035_S_4783	v03	099_S_4076	v02	141_S_1052	m48
014_S_4615	v03	035_S_4784	v01	099_S_4086	v02	141_S_1255	m48
016_S_0702	m48	035_S_4785	v02	099_S_4202	v01	141_S_1378	m36
016_S_1117	m36	035_S_6200	scmri	099_S_4205	v01	141_S_2210	sc
016_S_1326	m36	035_S_6480	sc	099_S_4480	v01	141_S_2333	sc
016_S_2284	sc	035_S_6488	scmri	099_S_4498	v01	141_S_4053	v01
016_S_4097	v02	035_S_6551	scmri	099_S_4994	v03	141_S_4160	v01
016_S_4121	v02	036_S_4389	v02	099_S_6016	scmri	141_S_4232	v01
016_S_4353	v03	036_S_4430	v01	099_S_6025	scmri	141_S_4426	v01
016_S_4583	v03	036_S_4491	v02	099_S_6038	scmri	141_S_4438	v01
016_S_4591	v03	036_S_4538	v01	099_S_6097	scmri	141_S_4456	v01
016_S_4688	v02	036_S_4714	v01	099_S_6175	scmri	141_S_4711	v01
016_S_4887	v03	036_S_4820	v03	099_S_6396	scmri	141_S_4803	v01
016_S_4902	v01	036_S_4878	v02	100_S_0047	m60	141_S_4907	v01
016_S_4951	v02	036_S_4894	v03	100_S_0069	m60	141_S_4976	v01
016_S_4952	v02	036_S_4899	v01	100_S_1286	m48	153_S_4077	v01
016_S_5007	v01	036_S_5063	v03	100_S_4556	v21	153_S_4125	v02
016_S_5032	v03	036_S_5112	v03	100_S_5075	v02	153_S_4133	v01
016_S_5251	v03	036_S_5210	v03	100_S_5096	v02	153_S_4139	v02
018_S_0055	v11	036_S_5271	v02	100_S_5102	v02	153_S_4151	v02
018_S_0142	v11	036_S_5283	v02	100_S_5106	v03	153_S_4159	v01
018_S_2133	sc	036_S_6088	scmri	109_S_2200	sc	153_S_4297	v01
018_S_2155	sc	036_S_6134	sc	109_S_4499	v02	153_S_4372	v02
018_S_2180	sc	036_S_6189	scmri	114_S_0166	v11	153_S_4621	v01
018_S_4313	v02	036_S_6316	scmri	114_S_0173	v06	153_S_4838	v01
018_S_4349	v02	037_S_0150	v06	114_S_0378	v06	153_S_5261	v02
018_S_4399	v02	037_S_0377	init	114_S_0416	v06	153_S_5267	v02
018_S_4400	v02	037_S_0552	v06	114_S_1106	v06	153_S_6274	sc
018_S_4696	v03	037_S_4001	v03	114_S_1118	v06	153_S_6336	sc
018_S_4809	v01	037_S_4015	v01	114_S_2392	v11	153_S_6450	sc
018_S_4868	v01	037_S_4030	v01	114_S_5047	v01	168_S_6049	scmri
018_S_4889	v01	037_S_4071	v41	114_S_5234	v02	168_S_6062	scmri
018_S_6207	scmri	037_S_4146	v01	114_S_6057	scmri	168_S_6065	scmri
018_S_6351	scmri	037_S_4214	v01	114_S_6063	scmri	168_S_6085	scmri
019_S_4252	v03	037_S_4750	v01	114_S_6113	scmri	168_S_6180	sc
019_S_4293	v01	037_S_4750	v21	114_S_6251	scmri	168_S_6233	scmri

019_S_4477	v03	037_S_4770	v03	116_S_0361	v06	168_S_6318	scmri
019_S_4548	v01	037_S_4879	v03	116_S_0657	m48	168_S_6350	scmri
019_S_4549	v03	041_S_0679	v06	116_S_1232	m48	168_S_6371	scmri
019_S_4680	v01	041_S_1010	m48	116_S_4010	v02	168_S_6413	scmri
019_S_4835	v02	041_S_1418	v06	116_S_4043	v02	168_S_6426	sc
019_S_5012	v03	041_S_4014	v02	116_S_4092	v02	168_S_6467	sc
019_S_5019	v03	041_S_4041	v02	116_S_4175	v01	177_S_6328	scmri
020_S_5140	v02	041_S_4060	v02	116_S_4195	v03	177_S_6408	scmri
020_S_5203	v02	041_S_4138	v01	116_S_4209	v03	301_S_6224	scmri
020_S_6185	scmri	041_S_4143	v01	116_S_4483	v02	301_S_6297	sc
021_S_0159	m60	041_S_4200	v02	116_S_4625	v03	301_S_6326	sc
021_S_0276	v11	041_S_4510	v01	116_S_4635	v01	941_S_1195	m48
021_S_0337	v11	041_S_4720	v01	116_S_4855	v02	941_S_1202	v06
021_S_0984	m48	041_S_4876	v01	123_S_0113	m60	941_S_2060	sc
021_S_2100	sc	041_S_4877	v01	123_S_1300	m48	941_S_4036	v01
021_S_2124	sc	041_S_4974	v01	123_S_2055	sc	941_S_4066	v02
021_S_2125	sc	041_S_4989	v01	123_S_4127	v01	941_S_4100	v02
021_S_2150	sc	041_S_5078	v02	123_S_4170	v01	941_S_4187	init
021_S_4245	v01	041_S_5082	v02	123_S_4526	v03	941_S_4365	v02
021_S_4254	v02	041_S_5100	v02	123_S_4904	v01	941_S_4376	v02
021_S_4276	v02	041_S_5141	v02	123_S_6118	scmri	941_S_4764	v01
021_S_4335	v02	041_S_5204	v02	126_S_0680	m60	941_S_5124	v02
021_S_4402	v01	041_S_5244	v02	126_S_0709	m60	941_S_5193	v02
021_S_4419	v01	041_S_6292	scmri	126_S_1187	v06		
021_S_4421	v02	041_S_6314	scmri	126_S_2405	sc		
021_S_4558	v02	041_S_6354	scmri	126_S_2407	sc		

OASIS database (Subject ID: **Sub_ID,** Day for PET: **PET,** Day for MRI: **MRI)**

Sub_ID	PET	MRI	Sub_ID	PET	MRI	Sub_ID	PET	MRI	Sub_ID	PET	MRI
OAS30001	2181	2430	OAS30272	3004	3087	OAS30585	0	65	OAS30867	4275	4407
OAS30002	2263	2340	OAS30276	2342	2405	OAS30586	733	749	OAS30875	1158	1252
OAS30003	2630	2682	OAS30291	1901	1979	OAS30589	2996	3191	OAS30881	3048	3163
OAS30004	3458	3457	OAS30293	1201	1221	OAS30594	0	66	OAS30887	1258	1407
OAS30006	2267	2342	OAS30296	0	69	OAS30597	3307	3137	OAS30896	3460	3528
OAS30007	1559	1641	OAS30304	0	27	OAS30601	1104	1146	OAS30907	3267	3359
OAS30010	0	68	OAS30306	507	473	OAS30603	2359	2253	OAS30910	964	1028
OAS30025	2247	2298	OAS30307	2264	2362	OAS30607	0	117	OAS30913	1930	2007
OAS30026	0	129	OAS30318	3226	3298	OAS30608	3928	3984	OAS30917	0	53
OAS30028	1190	1260	OAS30320	1886	1740	OAS30620	2183	2211	OAS30921	2359	2468
OAS30039	0	103	OAS30324	866	959	OAS30637	0	79	OAS30927	0	145
OAS30042	0	67	OAS30328	379	470	OAS30644	0	136	OAS30938	452	249
OAS30044	0	61	OAS30333	1927	2005	OAS30659	1582	1668	OAS30939	1856	1968
OAS30046	1869	1968	OAS30335	3679	3812	OAS30663	0	51	OAS30943	391	295
OAS30048	3321	3375	OAS30336	402	244	OAS30664	3429	3557	OAS30945	0	155
OAS30050	1418	1530	OAS30341	0	25	OAS30667	481	502	OAS30950	0	63
OAS30052	2650	2737	OAS30346	1595	1685	OAS30670	0	83	OAS30959	2594	2692
OAS30057	0	75	OAS30348	0	77	OAS30671	3437	3613	OAS30966	3316	3381
OAS30065	1923	2009	OAS30349	3204	3241	OAS30676	3531	3577	OAS30974	831	901
OAS30066	1837	2006	OAS30350	1158	1201	OAS30679	0	91	OAS30978	0	59
OAS30071	0	18	OAS30361	3183	3275	OAS30680	6124	6255	OAS30979	367	435
OAS30073	4455	4456	OAS30364	0	110	OAS30681	0	154	OAS30981	0	74
OAS30075	0	148	OAS30367	3338	3396	OAS30683	406	528	OAS30982	1574	1708
OAS30080	1262	1318	OAS30368	2289	2750	OAS30685	1397	1552	OAS30993	847	920
OAS30083	3062	2882	OAS30369	6896	6992	OAS30691	0	56	OAS30996	4581	4705
OAS30089	0	1	OAS30376	0	82	OAS30695	0	104	OAS30997	1113	1356
OAS30093	0	56	OAS30382	1136	1199	OAS30706	0	60	OAS31000	0	72
OAS30097	2370	2551	OAS30385	0	112	OAS30708	0	71	OAS31005	1557	1649
OAS30102	0	24	OAS30386	0	55	OAS30711	3286	3480	OAS31011	0	89
OAS30105	0	56	OAS30387	3358	3401	OAS30722	1112	1162	OAS31012	3896	4024
OAS30108	1981	2066	OAS30392	3086	3145	OAS30723	2541	2568	OAS31013	0	67
OAS30109	2237	2320	OAS30402	1064	1085	OAS30725	2355	2456	OAS31014	3331	3555
OAS30117	4050	4155	OAS30403	2277	2378	OAS30728	426	516	OAS31018	0	41
OAS30122	0	136	OAS30407	2687	2862	OAS30729	6092	6253	OAS31019	1298	1370
OAS30123	0	122	OAS30414	0	37	OAS30733	4940	4997	OAS31020	0	84
OAS30127	0	110	OAS30417	60	93	OAS30735	3303	3515	OAS31021	1262	1318
OAS30129	0	55	OAS30422	5	104	OAS30739	0	51	OAS31022	0	89
OAS30134	1487	1642	OAS30423	2253	2099	OAS30746	0	35	OAS31028	0	58
OAS30135	2263	2367	OAS30434	0	54	OAS30748	3138	3268	OAS31031	4173	4072
OAS30139	1605	1702	OAS30438	2234	2358	OAS30750	2263	2350	OAS31039	1182	1184
OAS30143	3359	3509	OAS30455	4125	4209	OAS30759	0	63	OAS31041	1162	1426
OAS30146	2190	2309	OAS30458	0	63	OAS30764	0	55	OAS31042	3477	3618
OAS30149	1164	1240	OAS30468	0	69	OAS30766	0	147	OAS31048	2272	2398
OAS30151	0	64	OAS30475	0	62	OAS30767	1019	948	OAS31056	1878	1912

OAS30155	720	785	OAS30476	420	482	OAS30768	2233	2337	OAS31068	3337	3484
OAS30159	0	110	OAS30479	2306	2421	OAS30769	1496	1547	OAS31070	0	84
OAS30178	2305	2455	OAS30483	0	20	OAS30770	1194	1210	OAS31071	0	68
OAS30182	0	101	OAS30484	1056	557	OAS30775	2284	2395	OAS31076	0	71
OAS30184	3065	3157	OAS30485	0	84	OAS30776	3896	4024	OAS31080	0	183
OAS30185	4485	4551	OAS30491	0	74	OAS30777	4624	4740	OAS31088	3106	3212
OAS30187	0	70	OAS30508	0	42	OAS30788	2960	2927	OAS31090	3461	3565
OAS30194	8827	8874	OAS30516	4089	4192	OAS30808	3293	3453	OAS31110	2223	2336
OAS30197	0	115	OAS30537	1596	1414	OAS30810	1240	1351	OAS31111	3501	3618
OAS30204	0	33	OAS30538	0	105	OAS30818	1875	1720	OAS31113	827	881
OAS30206	2939	3024	OAS30546	526	530	OAS30819	753	572	OAS31114	2610	2658
OAS30219	1183	1184	OAS30551	1207	1310	OAS30821	0	68	OAS31115	368	466
OAS30220	1066	1165	OAS30558	4376	4493	OAS30823	2549	2722	OAS31117	0	73
OAS30221	1079	1143	OAS30561	0	106	OAS30824	0	77	OAS31118	1966	2041
OAS30225	405	482	OAS30562	3340	3383	OAS30832	2305	2369	OAS31125	2953	3093
OAS30228	0	90	OAS30567	0	40	OAS30841	3447	3499	OAS31127	2063	2140
OAS30233	5189	5295	OAS30568	1190	1284	OAS30842	402	526	OAS31149	0	61
OAS30240	3406	3487	OAS30574	1841	1917	OAS30845	1162	1266	OAS31150	1315	1416
OAS30248	1144	1191	OAS30579	2372	2400	OAS30852	6873	6963	OAS31158	2354	2481
OAS30264	395	466	OAS30580	1468	1531	OAS30858	1890	2100	OAS31161	0	56
OAS30270	0	70	OAS30584	0	96	OAS30861	1605	1702	OAS31165	1072	1122
